# Efficacy and Safety Outcomes for Acute Ischemic Stroke Patients Treated with Intravenous Infusion of Tirofiban After Emergent Carotid Artery Stenting

**DOI:** 10.1007/s00062-023-01350-7

**Published:** 2023-10-05

**Authors:** Rana Garayzade, Ansgar Berlis, Stefan Schiele, Michael Ertl, Hauke Schneider, Gernot Müller, Christoph J. Maurer

**Affiliations:** 1 Department of diagnostic and interventional Radiology and Neuroradiology, Augsburg University Hospital, Augsburg, Germany; 2 Institute of Mathematics, Augsburg University, Augsburg, Germany; 3 Department of Neurology, Augsburg University Hospital, Augsburg, Germany

**Keywords:** Acute ischemic stroke, Endovascular treatment, Tirofiban, NIHHS score, Thrombolysis, Emergency carotid artery stenting, Tandem occlusion

## Abstract

**Introduction:**

Emergent stenting of the extracranial internal carotid artery (ICA) in stroke patients requires antiplatelet therapy to prevent in-stent thrombosis with a higher risk of intracranial haemorrhage.

**Aim of the Study:**

Assess the efficacy and safety of emergent carotid stenting with intravenous tirofiban in acute ischemic stroke patients.

**Methods:**

Primary endpoint: symptomatic hemorrhage. Secondary endpoints: 90-day functional outcome and mortality.

**Results:**

Of the 62 patients, 21 (34%) received tirofiban as a single antiplatelet, and 41 (66%) received combined therapy. Premedication with anticoagulants and antiplatelets was significantly more frequent in the tirofiban-only group. The rate of symptomatic haemorrhage was significantly lower in the tirofiban-only group than in the combined group (4.8% vs. 27%, *p* = 0.046). The patients with tirofiban alone had a significantly better functional outcome at day 90 than the combined group (52% vs. 24%, *p* = 0.028). Mortality was equal (24%) in both groups. Pre-interventional NIHSS score (*p* = 0.003), significant blood pressure fluctuations (*p* = 0.012), tandem occlusion (*p* = 0.023), and thrombolysis (*p* = 0.044) showed relevant influence on the rate of symptomatic hemorrhage in the entire patient cohort.

**Conclusions:**

A single antiplatelet therapy with tirofiban regardless of the premedication may improve the functional outcome in patients with stroke due to acute extracranial carotid lesion and emergent carotid stenting with lower rates of serious intracranial haemorrhage.

For patients with high pre-interventional NIHSS score, tandem occlusion and after pre-interventional thrombolysis, caution is advised. Additionally, strict blood pressure monitoring should be conducted during the first 72 h after intervention.

## Introduction

Acute ischaemic stroke (AIS) is a severe and life-threatening disease, especially when caused by large-vessel occlusion (LVO) [1].

Acute ischaemic strokes due to large vessel occlusion of the anterior circulation with concomitant ipsilateral extracranial internal carotid artery high-grade stenosis or occlusion (tandem occlusion, TO) account for up to 25% of patients considered for endovascular therapy [2, 3].

This patient population has a particularly worse prognosis without endovascular treatment [4]. The ideal endovascular treatment of acute cerebral large vessel occlusions and concomitant atherosclerotic lesions of the ipsilateral internal carotid artery (ICA) is still a matter of debate [5]. Recent retrospective multicentre studies have shown good results with simultaneous treatment of both occlusions with a good outcome (mRS 0–2) in up to 55% of patients [6–8]. Furthermore, a pooled analysis of the retrospective international TITAN trial and the prospective multicenter observational ETIS registry showed that acute cervical ICA stenting is associated with higher odds of favourable clinical outcome and successful reperfusion in patients with ischaemic strokes due to TO [9].

Carotid artery stenting requires antiplatelet therapy for the prevention of in-stent thrombosis [10]. However, there is currently no consensus on the ideal antithrombotic medication for patients with acute ischaemic stroke due to anterior circulation TOs treated with emergent carotid stent placement [11]. The antithrombotic treatment strategies in this group of patients vary widely from centre to centre [12–15].

Recent data suggest that tirofiban is a safe and effective alternative in neuroradiological interventions [16, 17].

However, data are still scarce; therefore, the principal aim of this study was to investigate the efficacy and safety of emergency carotid artery stenting in a stroke patient population treated with intravenous tirofiban.

## Material and Methods

### Patient Population

The study involved 62 patients who were treated between 2010 and 2019 at our Neurovascular Center for acute ischaemic stroke with a total occlusion or high-grade stenosis of the extracranial ICA of at least 90% by NASCET criteria. The patients were treated with emergent stenting and concomitant intravenous tirofiban infusion. Patients without intracranial occlusions were also included in the study.

### CT Examination

All patients underwent multimodal computed tomography (CT), which included noncontrast-enhanced CT and CT-angiography (CTA). CT perfusion was additionally performed in the patients with wake-up stroke, an unclear onset time or who presented later than 4.5 h after they were last seen to be normal.

### Administration of Tirofiban

We used a high-dose tirofiban bolus regimen adopted from percutaneous coronary interventions in cardiology [18]. Tirofiban was administered intravenously as a bolus of 25 mcg/kg within 3 min during stenting followed by an infusion of 0.15 mcg/kg/min, and this continued for up to 24 h after the procedure, when no ICH or subarachnoid haemorrhage (SAH) was found on the CT scans that were performed in the angio suite immediately after the procedure. The tirofiban dose was reduced by 50% in the patients with severe renal insufficiency (eGFR < 30 ml/min). If an earlier follow-up CT showed significant ICH or severe systemic bleeding in case of clinical deterioration, the tirofiban infusion was discontinued. Administration of additional medications such as ASA or anticoagulants was at the discretion of the treating physician and depended mainly on the premedication known at the time of emergency treatment.

### Blood Pressure Management

Careful blood pressure management is critical in the post-stenting period to optimize patient outcomes and to avoid hyperperfusion syndrome and bleeding complication. Strict monitoring of blood pressure parameters below 140 mmHg was recommended in the stroke unit.

### Outcomes

The primary outcome parameter was the occurrence of symptomatic ICH (parenchymal haemorrhage accompanied by neurological deterioration of ≥ 4 points on the National Institutes of Health Stroke Scale, NIHHS). The secondary outcome measures were the rate of good clinical outcome (modified Rankin Scale mRS of 0–2) and mortality, which were both determined at 3 months.

### Statistics

Descriptive statistics were used to summarize the patient characteristics and outcomes. Continuous variables were described by the median and interquartile range. Categorical variables were expressed as numbers and percentages. Variables were compared with Wilcoxon rank-sum tests for continuous variables and a chi-squared test or Fisher exact tests for categorical variables. To investigate the association between the outcomes and characteristics, we performed a logistic regression analysis for each outcome. Further, we performed a multivariable logistic regression for each outcome with all variables that were deemed significant in the univariable regression. To ensure the number of variables is not too high, these models were reduced in variable size with a stepwise algorithm according to their AIC.

All statistical analyses were performed with R 4.1.1. A *p*-value *p* < 0.05 was considered statistically significant.

## Results

### Study Patients and Baseline Characteristics.

A total of 62 patients were included in the analyses.

Forty-seven (76%) patients had tandem occlusion with proximal intracranial occlusion (distal ICA, M1or M2) and cervical carotid artery lesions. The baseline patient characteristics are displayed in Table 1. The patients were divided into two groups: the first group was comprised of 41 (66%) patients who received tirofiban in combination with other antiplatelets and/or anticoagulants and the second group was comprised 21 (34%) patients who received tirofiban alone.

**Table 1:** Baseline characteristics, peri-interventional imaging, and procedural characteristics

Characteristic	Overall, *N* = 62	Combined tirofiban group, *N* = 41	Tirofiban only, *N* = 21	*p*-value
*Age Mean (SD)*	67.6 (11.7%)	67.6 (13.0%)	67.6 (9.1%)	> 0.9
*rt-PA*				
Yes	37 (60%)	28 (68%)	9 (43%)	0.053
No	25 (40%)	13 (32%)	12 (57%)	
*Sex*				
m	37 (60%)	22 (54%)	15 (71%)	> 0.9
w	25 (40%)	19 (46%)	6 (29%)	
*NIHSS score*				
Median (25–75%)	14.5 (5.3–19.0)	15.0 (9.0–19.0)	10.0 (2.0–18.0)	> 0.9
Mean (SD)	13.0 (7.5)	14.5 (7.1)	10.1 (7.7)	
*mRS baseline*				
1	3 (4.8%)	1 (2.4%)	2 (9.5%)	> 0.9
2	6 (9.7%)	2 (4.9%)	4 (19%)	
3	4 (6.5%)	3 (7.3%)	1 (4.8%)	
4	18 (29%)	14 (34%)	4 (19%)	
5	31 (50%)	21 (51%)	10 (48%)	
*Aspects score baseline*				
6	1 (1.6%)	1 (2.4%)	0 (0%)	> 0.9
7	5 (8.1%)	2 (4.9%)	3 (14%)	
8	14 (23%)	9 (22%)	5 (24%)	
9	18 (29%)	11 (27%)	7 (33%)	
10	24 (39%)	18 (44%)	6 (29%)	
*Occlusion side*				
Left	35 (56%)	25 (61%)	10 (48%)	> 0.9
Right	27 (44%)	16 (39%)	11 (52%)	
*Tandem occlusion*				
Yes	47 (76%)	34 (83%)	13 (62%)	0.067
No	15 (24%)	7 (17%)	8 (38%)	
*Premedication*				
ASA	19 (31%)	7 (17%)	12 (57%)	< 0.001
ASA + Clopidogrel	3 (5%)	0	3 (14%)	
ASA + Clopidogrel + Heparin	1 (1.5%)	0	1 (5%)	
No previous medication	38 (61%)	33 (81%)	5 (24%)	
Previous therapy not known	1 (1.5%)	1 (2%)	0	
*Distal ICA, M1, M2*				
Distal ICA	6 (9.7%)	4 (9.8%)	2 (9.5%)	> 0.9
M1	31 (50%)	20 (49%)	11 (52%)	
M2	12 (19%)	12 (29%)	0 (0%)	
No	13 (21%)	5 (12%)	8 (38%)	
*TICI Score*				
1	2 (3.2%)	1 (2.4%)	1 (4.8%)	> 0.9
2a	3 (4.8%)	2 (4.9%)	1 (4.8%)	
2b	33 (53%)	23 (56%)	10 (48%)	
3	24 (39%)	15 (37%)	9 (43%)	
*ASPECTS post-interventional*				
5	5 (8.1%)	3 (7.3%)	2 (9.5%)	> 0.9
6	10 (16%)	6 (15%)	4 (19%)	
7	18 (29%)	13 (32%)	5 (24%)	
8	8 (13%)	4 (9.8%)	4 (19%)	
9	18 (29%)	15 (37%)	3 (14%)	
10	3 (4.8%)	0 (0%)	3 (14%)	
*Thrombectomy technique*				
Only Aspiration	3 (4.8%)	1 (2.4%)	2 (9.5%)	> 0.9
No thrombectomy	19 (31%)	11 (27%)	8 (38%)	
Solumbra	38 (61%)	28 (68%)	10 (48%)	
Stent retriever	2 (3.2%)	1 (2.4%)	1 (4.8%)	
*Time from groin punction to final recanalization (min)*				
Median (25–75%)	65.0 (61.0–75.0)	68.0 (63.0–75.0)	62.0 (57.0–69.0)	> 0.9
Mean (SD)	71.1 (21.1)	70.1 (12.0)	73.0 (32.6)	
*Time from onset to recanalization (min)*				
Median (25–75%)	290.5 (217.8–356.5)	291.0 (211.0–355.0)	289.0 (220.5–369.0)	> 0.9
Mean (SD)	297.7 (99.1)	295.1 (91.8)	302.2 (113.1)	
Unknown	10	8	2	
*Procedural complications*				
Yes	2 (3.2%)	1 (2.4%)	1 (4.8%)	> 0.9
No	60 (97%)	40 (98%)	20 (95%)	
*Number of thrombectomy maneuvers*				
Median (25–75%)	1.0 (0.0–2.0)	1.0 (0.0–2.0)	1.0 (0.0–2.0)	> 0.9
Mean (SD)	1.4 (1.4)	1.4 (1.2)	1.4 (1.7)	
*Time of occurrence of the hemorrhage (hour)*				
Median (25–75%)	6.0 (3.8–7.3)	6.0 (3.8–7.3)	6.0 (4.5–7.3)	> 0.9
Mean (SD)	5.3 (3.3)	5.3 (3.5)	5.1 (3.3)	
Unknown	38	25	13	
*Relevant blood pressure fluctuations*				
Yes	11 (18%)	8 (20%)	3 (14%)	> 0.9
No	51 (82%)	33 (80%)	18 (86%)	

In the combined group, more patients (68%) received rt-PA prior to intervention than in the only tirofiban group (43%), but the difference was not significant (*p* = 0.053).

Twenty-three (37%) patients in our cohort were on antiplatelet or anticoagulant medication upon admission. Among the patients who received peri-interventional tirofiban as a single antiplatelet, 76% already had at least one antiplatelet in their previous medication. In contrast, 81% of the patients in the combined group had no known premedication. Hence, there was a significant difference between the two groups in terms of premedication.

There were no significant differences between the two groups in terms of remaining pre-/post-interventional imaging findings, pre-interventional patient status, procedure duration, and technical success (TICI score), as shown in the Table 1.

Tirofiban was used in 46 (46/62, 74%) patients shortly before stent implantation. In 12 (12/62, 19%) patients, tirofiban was given after stent implementation and after detection of in-stent thrombosis during the control angiography. In 4 patients (4/62, 6.5%), tirofiban was given after detection of distal embolization during the control angiography after stent implantation.

The combination variants of tirofiban with antiplatelet/anticoagulant are shown in Table 2. The choice of further antithrombotic therapy was left to the discretion of the treating physicians.

**Table 2:** Group with combined antithrombotic treatment (*N* = 41)

Tirofiban plus ASA 500 mg	Tirofiban plus ASA 100 mg	Tirofiban plus Heparin 2500 IE	Tirofiban plus Heparin 5000 IE	Tirofiban plus ASA 100 mg plus Heparin 2500 IE	Tirofiban plus ASA 500 mg plus Heparin 5000 IE
15 (36.6%)	18 (43.9%)	2 (4.9%)	3 (7.3%)	1 (2.4%)	2 (4.9%)

## Safety and Clinical Outcomes

The safety and clinical outcomes are shown in Table 3.

**Table 3:** Safety and clinical outcomes

Characteristic	Total cohort (*n* = 62)	Tirofiban group (*n* = 21)	Combined group (*n* = 41)	*p*-value
Symptomatic haemorrhage	12 (19%)	1 (4.8%)	11 (27%)	0.046
mRS 0–2 at 3 months	21 (34%)	11 (52%)	10 (24%)	0.028
Mortality at 3 months	15 (24%)	5 (24%)	10 (24%)	> 0.9

### Safety and Clinical Outcome in Our Total Cohort

Nineteen percent of the patients in our overall cohort suffered from symptomatic haemorrhage, 34% of the patients had a good functional outcome, and 24% of the patients died (see Table 3).

We observed serious thrombocytopenia (platelet count < 50 × 10^9/L) in 2 cases (2/62, 3.2%) in our study. Both of the patients who had serious thrombocytopenia in the combined group were treated with a combination of tirofiban and ASA 100 mg. Both patients died; one patient died from haemorrhagic shock due to diffuse haemorrhage, and one patient died from massive intracranial haemorrhage.

### Primary Endpoint by Subgroup Analysis: Serious Haemorrhage

Our analysis revealed a statistically significant difference between the two treatment groups with regard to the rate of serious haemorrhage (4.8% in the tirofiban group versus 27% in the combined group, *p* = 0.046), as shown in Table 3.

### Secondary Endpoints by Subgroup Analysis: Good Functional Outcome and Mortality at 90 Days

The rate of good functional outcome at 3 months (mRS 0–2) was significantly higher in the tirofiban group than in the combined group (52% vs. 24%, *P* = 0.028).

There was no statistically significant difference between the two treatment groups in mortality (24% in each group, *p* > 0.9).

### Multivariable Logistic Regression Analysis for Predictors of Symptomatic Hemorrhage, Good Outcome and Mortality

The multivariate analyses revealed significant effects of certain factors mentioned in Table 1 on the rates of intracranial hemorrhage, outcomes, and mortality for the entire patient cohort (Table 4). Pre-interventional NIHSS score (*p* = 0.003), significant blood pressure fluctuations (*p* = 0.012), tandem occlusion (*p* = 0.023), and thrombolysis (*p* = 0.044) showed relevant influence on the rate of symptomatic hemorrhage. Factors such as pre-interventional ASPECTS score (*p* = 0.046), time from symptom onset to recanalization (*p* = 0.029), and time from groin puncture to final recanalization (*p* = 0.019) demonstrated significant impact on favorable outcomes (mRS at 3 months). The pre-interventional NIHSS score significantly influenced mortality (*p* = 0.002).

**Table 4:** Multivariable logistic regression analysis for predictors of symptomatic hemorrhage, good outcome and mortality

	Estimate	*p*-value
–	*Mortality*	
NIHSS preinterventional	1.454 (1.205–1.946)	0.002
Sex:w, ref m	0.121 (0.007–1.056)	0.089
Intravenous alteplase treatment:yes, ref no	0.236 (0.034–1.304)	0.11
–	*mRS_0–2_at_3_months*	
Aspects score preinterventional	2.424 (1.064–6.369)	0.046
Number of thrombectomy maneuvers	2.483 (0.870–9.033)	0.117
Time from onset to rekanalisation(min)	0.987 (0.974–0.998)	0.029
Time from punction to final rekanalisation(min)	0.852 (0.732–0.959)	0.019
–	*Symptomatic_hemorrhage*	
NIHSS preinterventional	1.581 (1.248–2.320)	0.003
Significant blood pressure fluctuations:yes, ref no	88.468 (4.622–6733.877)	0.012
Tandem_Occlusion:yes, ref no	537.026 (7.324–558,771.866)	0.023
Intravenous alteplase treatment:yes, ref no	0.062 (0.002–0.687)	0.044

### Stent Patency

Ultrasound, performed within 1–2 days after stenting, showed that the carotid stent was open in 35 of the 36 patients with available ultrasound data in our cohort (97%). Stent occlusion was observed in 1 patient (2.8%).

Follow-up ultrasound data were not available for 26 patients, making it difficult to compare the two groups in terms of stent occlusion.

Dual antiplatelet therapy was administered after the follow-up CT at 24 (+/−6) hours, and tirofiban was substituted for ticagrelor or clopidogrel after stopping the tirofiban infusion in 9 patients (9/62, 14.5%), with overlapping in 40 patients (40/62, 64.5%), mostly by using ASA and ticagrelor.

A single antiplatelet was administered in 3 patients (3/62, 4.8%), and no medication was administered in 10 patients (10/62, 16.1%). A multiplate test was used to check for non-responders to clopidogrel or ticagrelor.

### Blood Pressure Management

Target values of blood pressure of 140 mmHg were achieved in 51 patients (82%). In 11 patients (18%), significant blood pressure fluctuations were observed, with blood pressure dropping below 60 mmHg and blood pressure rising above 185 mmHg within 72 h after stenting. In cases of blood pressure drop, catecholamines were administered, and for blood pressure reduction, urapidil was administered.

5 patients (all from the combined group) had hypertensive blood pressure values. 2 patients (all from the tirofiban alone group) had hypotensive blood pressure values below 60 mmHg. 3 patients (from the combined group) initially had hypotensive values, and under the influence of catecholamines, hypertensive values were observed. In 1 patient from the tirofiban alone group, the situation was reversed—initially hypertensive, and under urapidil administration, hypotensive blood pressure values were observed.

All patients who exhibited hypotensive values also showed progressive infarct demarcation within the first 72 h after stenting, with a decrease in ASPECTS score by over 2 units, despite good results from thrombectomy (Table 5).

**Table 5:** Relevant blood pressure fluctuations

	*Combined group*	Outcome	Symptomatic haemorrhage
1	Hypertensiv	mRS 3–5	No symptomatic haemorrhage
2	Hypotensiv/hypertensiv	mRS 3–5	Intracerebral hemorrhagic malignant infarction
3	Hypertensiv	mRS 3–5	No symptomatic haemorrhage
4	Hypotensiv/Hypertensiv	mRS 6	Intracerebral hemorrhagic malignant infarction
5	Hypertensiv	mRS 6	Symptomatic haemorrhage
6	Hypertensiv	mRS 3–5	Symptomatic haemorrhage
7	Hypertensiv	mRS 3–5	No symptomatic haemorrhage
8	Hypotensiv/hypertensiv	mRS 6	Symptomatic haemorrhage
–	*Tirofiban alone*	–	–
9	Hypertensiv/hypotensiv	mRS 6	No symptomatic haemorrhage
10	Hypotensiv	mRS 3–5	No symptomatic haemorrhage
11	Hypotensiv	mRS 3–5	No symptomatic haemorrhage

## Discussion

Our data show that patients with severe ischaemic stroke due to acute ICA occlusion treated with tirofiban infusion as the sole antiplatelet therapy in the setting of emergency carotid stenting had a better functional outcome with lower rates of severe intracranial haemorrhage than patients receiving tirofiban in combination with other antiplatelet agents and/or anticoagulants. It should be noted that 76% of the patients who received periinterventional tirofiban alone already had at least one antiplatelet in their previous medication.

Steno-occlusion of the cervical internal carotid artery (ICA) poses a technical challenge in the treatment of TO by endovascular means [6]. Emergent stenting of the extracranial carotid lesion is a predictor of successful reperfusion and favourable outcome in this complex patient population [19–21].

Following the procedure, there is a risk of thromboembolic complications due to platelet activation and aggregation on the surface of the stent. The optimal antiplatelet/anticoagulant management to 1) reduce the risk of postinterventional symptomatic intracranial haemorrhage (sICH) and 2) prevent stent reocclusion in these patients remains controversial [11].

We used tirofiban in all patients in our cohort. The fast-acting and fast-deactivated drug with a short half-life (approximately 2 h) antagonizes platelet glycoprotein IIb/IIIa (GP IIb/IIIa) receptors. Since the binding of fibrinogen to this receptor is a necessary step for normal platelet aggregation with any inducer, GP IIb/IIIa receptor inhibiting properties are regarded as targets in suppressing platelet function [22].

GP IIb/IIIa inhibitors prevent thrombus formation and thus avoid serious postprocedural complications of stent occlusion and subsequent distal embolization [23]. The large cardiological PRIMS-PLUS study did not report any case of ICH by using tirofiban within a sample of 1570 patients with acute myocardial infarction and unstable angina [24]. Thrombocytopenia during the administration was infrequent in this trial and was rapidly reversible without sequelae after the cessation of the infusion [24]. Severe thrombocytopenia with so-called vanishing platelets by using tirofiban is a rare complication [25]. In our study, we observed acute and severe thrombocytopenia in 2 patients (2/62, 3.2%). Both patients died from haemorrhagic complications. Monitoring platelet counts is important, particularly within 24 h after tirofiban administration, to detect most cases of acute thrombocytopenia [26]. If thrombocytopenia is identified and the infusion is stopped in time, supportive therapy with platelet transfusions is likely to be sufficient [27].

A meta-analysis found that tirofiban combined with EVT for patients with acute ischaemic stroke increased the incidence of favourable functional outcomes and did not increase the risk of sICH or mortality [28].

Our retrospective single-centre study also showed no increased risk of sICH by using tirofiban compared with other antiplatelet/anticoagulant agents, as described in other studies [8, 11, 12, 14]. Moreover, despite premedication with at least one antiplatelet agent in 76% of the patients in the subgroup with only tirofiban periinterventional therapy, the risk of sICH was relatively low, at 4.8%.

For comparison, the meta-analysis of the HERMES collaboration that pooled patient-level data from five randomized trials (MR CLEAN, ESCAPE, REVASCAT, SWIFT PRIME, and EXTEND IA) in 2015, showed in a patient population with AIS caused by LVO occlusion and treated with EVT a rate of symptomatic intracranial hemorrhage of 4.4% patients, a good outcome with mRS of 0–2 by 46% patients and a mortality rate of 15.3% [29]. These results are in line with our results in the only tirofiban group, although the rate of TO in the HERMES analysis was only 9.5% (122/1278). Even the results of the TITAN Registry for patients with TO with 5.9% sICH were comparable with our results of sICH of 4.8% by using tirofiban only [30].

The results of prospective multicenter observational registries showed that an aggressive antiplatelet regimen including oral or intravenous glycoprotein (GP) IIb/IIIa or P2Y12 inhibitors in tandem occlusions treated with CAS plus thrombectomy was associated with an increased rate of carotid stent patency at Day 1 without safety concerns compared to patients who received aspirin alone [17].

Brockmann et al. analysed the risk profile for the off-label use of tirofiban in interventional neuroradiology patients and concluded that the safety profile of tirofiban, when used off-label in a neuroendovascular setting, is acceptable but noted that the proportionally highest complication risk applies to older patients and patients being treated for acute stroke [16].

As our analysis demonstrates, a poor baseline NIHHS correlates with the rate of symptomatic hemorrhage (*p* = 0.003) and mortality (*p* = 0.002). In patients with a high pre-interventional NIHSS score, a compromised collateral situation and consequently a rapidly enlarging infarct core can be expected [31, 32]. It appears crucial to perform a flat-panel CT images in the angiography suite prior to initiating Tirofiban, whether administered alone or in combination with other anticoagulant/antiplatelet agents.

Multivariable regression analyses also demonstrated a significant impact of tandem lesions on the rate of symptomatic hemorrhage (*p* = 0.023).

Our results also demonstrate a significant impact of pre-interventional intravenous treatment with Alteplase on the rate of symptomatic bleeding. It it is still unclear whether it may be better to avoid rTPA administration when the likelihood of stenting is high and to start dual antiplate therapy already periinterventionelly, for example aspirin und tirofiban [33].

This aspect is of concern from the neurological point of view, as thrombolysis is the standard therapy for stroke, and forgoing thrombolysis is only recommended in cases of clear contraindications according to guidelines.

Such variations as time from onset to recanalization (*p* = 0.029) and time groin punction to final recanalization (*p* = 0.019) are statistically relevant for a favorable outcome. Primarily, this concerns patients with high initial NIHSS scores, who may develop large infarctions in a shorter period of time.

Blood pressure management after carotid artery stenting plays a critical role in the context of reperfusion injury [34].

Hypertension, or high blood pressure, following carotid artery stenting can exacerbate reperfusion injury by increasing cerebral blood flow beyond the capacity of the injured brain tissue. The high pressure can also lead to hemorrhagic transformation, particularly in the context of large infarcts.

On the other hand, hypotension can lead to inadequate perfusion and thus increase the extent of the ischemic insult, particularly in regions of the brain already at risk due to the initial ischemic event.

Therefore, maintaining an optimal blood pressure range is crucial to minimize reperfusion injury following carotid artery stenting. Current guidelines suggest maintaining systolic blood pressure below 140 mmHg in the immediate period after reperfusion therapies, although the optimal target may vary based on individual patient characteristics and clinical context [35].

Both hypertension and hypotension can exacerbate injury, and careful blood pressure management is critical in the post-stenting period to optimize patient outcomes.

In our study, 11 patients experienced significant blood pressure fluctuations within the first 72 h, correlating with poor outcomes and/or significant bleeding. Numerically, there were many more patients with significantly elevated blood pressure and symptomatic bleeding in the combined group, further emphasizing the importance of tight blood pressure control to prevent significant bleeding.

We therefore strongly recommend that all patients following stent placement should be closely monitored in an intensive care unit with invasive blood pressure measurement.

Current literature shows that emergent stenting appears to be an effective treatment strategy in patients with AIS due to LVO and extracranial lesions of ACI, although prospective randomized controlled trials are missing. The ongoing multicentre, prospective **EASI-TOC **Trial, for example, compares cervical emergent stenting to no stenting for tandem occlusion with immediate postprocedural single antiplatelet ASA. A second agent (usually clopidogrel 300 mg PO) is added after follow-up brain imaging at 12–24 h after confirming the absence of significant ICH.

Whether ASA or another agent, such as tirofiban, is the optimal choice in these patients remains to be decided, and further data in this regard are clearly necessary. However, the problem seems to be that many stroke patients already have ASA as premedication, and it is questionable whether additional intravenous administration of only ASA in such patients would produce better results than adding tirofiban as a second antiplatelet agent in addition to ASA in the premedication. The periinterventional administration of tirofiban combined with ASA with a higher risk of bleeding only seems necessary in the case of a complete lack of premedication, which is rare in stroke patients.

After acute stenting with Tirofiban administration, we switched most of patients to dual antiplatelet therapy with Aspirin and Ticagrelor.

On one hand, Clopidogrel is administered as a prodrug, and we must wait for it to become bioactive through hepatic metabolism [36], and then conduct a multiplate test. On the other hand, approximately one third of patients are non-responders to Clopidogrel, whereas non-responders to Ticagrelor are very rare [37]. As a result, administering Ticagrelor after stenting is less critical, as there is reduced uncertainty following the medication’s administration. Therefore, during the acute phase, taking Ticagrelor is reasonable despite higher costs, as it serves patient safety.

We acknowledge several limitations of our study: the retrospective single-centre design, relatively small cohort, lack of control group and rather inhomogeneous anticoagulant regimen.

However, our data still showed a significantly lower rate of sICH with comparable morbidity and mortality after 3 months with tirofiban alone even in patients premedicated with antiplatelet therapy, which is also often the case in stroke patients.

Moreover, although the use of tirofiban in the setting of acute carotid artery stenting shows promise, more large-scale, randomized clinical trials are needed to better define its role in this context. Therefore additional high quality research is needed.

## Conclusion

A single antiplatelet therapy with tirofiban regardless of premedication may improve the functional outcome in patients with stroke due to acute extracranial ICA occlusion or high-grade stenosis and emergent carotid stenting with lower rates of serious intracranial haemorrhage.

For patients with high pre-interventional NIHSS score, tandem occlusion and after pre-interventional thrombolysis, caution is advised. Additionally, strict blood pressure monitoring should be conducted during the first 72 h after intervention.

## Abbreviations

ASPECTS: Alberta Stroke Program Early CT Score
CT: Computed tomography
mRS: Modified Rankin score
NIHSS: National Institutes of Health Stroke Scale
rt-PA: recombinant tissue-type plasminogen activator
